# Radioactivity, Metals Pollution and Mineralogy Assessment of a Beach Stretch from the Ionian Coast of Calabria (Southern Italy)

**DOI:** 10.3390/ijerph182212147

**Published:** 2021-11-19

**Authors:** Francesco Caridi, Giuseppe Paladini, Valentina Venuti, Vincenza Crupi, Salvatore Procopio, Alberto Belvedere, Maurizio D’Agostino, Giuliana Faggio, Rossella Grillo, Santina Marguccio, Giacomo Messina, Domenico Majolino

**Affiliations:** 1 Dipartimento di Scienze Matematiche e Informatiche, Scienze Fisiche e Scienze della Terra, Università degli Studi di Messina, V.le F. Stagno D’Alcontres 31, 98166 Messina, Italy; fcaridi@unime.it (F.C.); dmajolino@unime.it (D.M.); 2 Dipartimento di Scienze Chimiche, Biologiche, Farmaceutiche e Ambientali, Università degli Studi di Messina, V.le F. Stagno D’Alcontres 31, 98166 Messina, Italy; vcrupi@unime.it; 3 Agenzia Regionale per la Protezione dell’Ambiente della Calabria (ARPACal), Dipartimento di Catanzaro, Via Lungomare (loc. Giovino), 88100 Catanzaro, Italy; s.procopio@arpacal.it; 4 Agenzia Regionale per la Protezione dell’Ambiente della Calabria (ARPACal), Dipartimento di Reggio Calabria, Via Troncovito SNC, 89135 Reggio Calabria, Italy; a.belvedere@arpacal.it (A.B.); m.dagostino@arpacal.it (M.D.); s.marguccio@arpacal.it (S.M.); 5 Dipartimento di Ingegneria dell’Informazione, delle Infrastrutture e dell’Energia Sostenibile (DIIES), Università Mediterranea, Loc. Feo di Vito, 89122 Reggio Calabria, Italy; gfaggio@unirc.it (G.F.); rossella.grillo@unirc.it (R.G.); messina@unirc.it (G.M.)

**Keywords:** radioactivity, radiological risk, heavy metals, pollution, mineralogy, sand

## Abstract

In the present article, a case study is reported regarding an investigation carried out in order to assess radioactivity concentration, heavy metals pollution and mineralogy of a beach stretch extending from Soverato to Squillace municipalities of the Ionian coast of Calabria, South of Italy, a popular tourist destination, especially in summer. The analysis of radionuclides contents was performed by using a High Purity Germanium (HPGe) gamma-ray detector, in order to quantify the average specific activity of ^226^Ra, ^232^Th and ^40^K natural radionuclides and ^137^Cs anthropogenic radioisotope. The absorbed dose rate and the annual effective dose equivalent radiological hazard indices were also estimated. Furthermore, X-ray Fluorescence (XRF) spectrometry measurements were carried out for the quantitative elemental analysis of the sand, in order to investigate any possible chemical pollution by heavy metals. For this aim, different indices such as Enrichment Factor (EF), Geoaccumulation Index (I_geo_), Contamination Factor (CF) and Pollution Load Index (PLI) were applied to estimate the level of toxicity imposed on the ecosystem by the detected heavy metals. Finally, in order to identify the crystalline mineral components of the investigated sand samples, X-ray Diffraction (XRD) and Micro-Raman Scattering (MRS) measurements were carried out.

## 1. Introduction

Environmental natural radioactivity, due to the presence of cosmogenic and primordial radionuclides in the Earth’s crust, provides the greatest contribution to the dose received by the population From the point of view of natural radioactivity, significant radionuclides are those belonging to ^238^U, ^232^Th, ^235^U radioactive chains and primordial ^40^K [1]. Together with their product of decay, these radionuclides are commonly found in environmental matrices, in particular in water, rocks and soils [2,3]. For the latter, the chemical composition and natural radioactivity widely vary with the geomorphological and geographical features of the investigated site. Beach sand deposits, in particular, are the result of erosion and weathering of metamorphic and igneous rocks, where the highest levels of natural radionuclides are found [4,5,6,7,8,9,10]. Thus, an investigation focused on the assessment of the specific activity of natural radioisotopes in beach sands allows to evaluate the radiological risk, due to the external gamma radiation exposure for individuals who spend their holidays on these beaches. This appears a crucial task since, as reported in the literature, [11,12,13], long-term exposure to uranium and thorium has several health effects, such as chronic lung diseases, acute leucopoenia, anaemia, and necrosis of the mouth; radium exposure causes bone, cranial, and nasal tumours; thorium exposure can cause lung, pancreas, hepatic, bone, and kidney cancers and leukaemia.

Furthermore, an alarming level of chemical pollutants is today present in many cities and coastal areas due to rapid industrialization and uncontrolled urbanization around these environments. Among these contaminants, heavy metals are of major concern for their persistent and bio-accumulative nature [14,15,16,17]. The knowledge of the sources of certain heavy metals and their contamination mechanisms of systems where their concentration levels reach toxicity levels is extremely important in addressing the extent of contamination itself [18]. Pollution of the natural environment by heavy metals is a universal problem, considering the toxic effects on living organisms when permissible concentration levels are exceeded [19]. Toxic metals may lead to a decline in the mental, cognitive and physical health of the individual [20]. According to [21], heavy metals occurrence in soils, waters and biota can be indicative of the presence of natural or anthropogenic sources.

Finally, geochemical studies of soils can be helpful in order to understand element distribution patterns and to evaluate the environmental conditions existing in a specific area [22]. In addition, the mineralogical properties of soils reflect the geological history of the transport and sorting process [23].

In the present study, a multidisciplinary approach—including the use of several analytical methods such as High Purity Germanium (HPGe) gamma-ray spectrometry, X-ray Fluorescence (XRF) Spectrometry, X-ray Diffractometry (XRD) and Micro-Raman Scattering (MRS)—was employed to assess the radioactivity and heavy metals contents and to relate them to the mineralogical and geochemical composition of the investigated beach stretch, extending from Soverato to Squillace in the Ionian coast of Calabria (Southern Italy), characterized by a peculiar reddish sand [24]. The choice of the investigated area was justified by the fact that, at the end of 2016, this stretch of beach was the centre of Italian media attention for an alleged contamination by artificial radionuclides, generated by the presence of hypothetical waste and/or radioactive materials. Calculations of the absorbed dose rate and of the annual effective dose equivalent were performed with the aim of assessing any possible radiological hazards for tourists or the inhabitant population. Different pollution indices, such as Enrichment Factor (EF), Geoaccumulation Index (I_geo_), Contamination Factor (CF) and Pollution Load Index (PLI) were also calculated in order to estimate the level of toxicity imposed on the ecosystem by heavy metals.

## 2. Geological Setting

The studied stretch of beach is placed along the Ionian coastline of the Calabria-Peloritani Arc (CPA). CPA is located in the centre of the Mediterranean, along the Apennine-Maghrebid chain, and it constitutes a distinct tectonic-stratigraphic domain [25], characterized by the presence of rocks, pre-alpine plutonic and metamorphic, associated with continental and oceanic metamorphites (Complex Ophiolitic Auct.), and tectonically superimposed on Mesozoic carbonate and flysch rocks [26]. Its tectonic evolution is part of the more general phenomenon of convergence of the African plates and European and has been the subject of various interpretative models, essentially attributable to two main groups. In some papers [27], the rocks of the Alpine chain present in Calabria (Austro-Alpine Unit) are part of the African plate involved in verging S-SSE subduction; in other works [28], the rocks of the Alpine chain are, on the contrary, a portion of the European margin, overrun on African units in the context of verging subduction towards NW. The generally accepted synthetic tectonic-stratigraphic scheme for the Arco-Calabro consists of an Apennine complex, an ophiolitic complex and the Calabride Auct. complex. In particular, many authors [25,26,29] report a tectonic-stratigraphic succession consisting of a series of superimposed units between the Oligocene and the Upper Miocene, grouped into units of the Apennine chain and units of the Alpine chain.

The investigated area falls into a geological environment characterized by two main sectors: the western one, in which the high-grade metamorphites of the Hercynian age are mainly diffused, and the eastern sector, in which the late Carboniferous granitoids emerge. The two sectors together constitute the part of the lower and intermediate continental crust of the late Carboniferous age which is found on the surface, and which is part of the Serre Massif. The latter in fact constitutes, together with the Sila Massif, the Coastal Range, the Peloritani, the Aspromonte Massif and Capo Vaticano (as well as Mount Sant’Elia), one of the structural elements of the CPA [25]. The investigated beach stretch falls in the central portion of the CPA, characterized by prevailing granitoid rocks (calcalkaline and peraluminous) with subordinate gneiss and phyllites [30,31]. The complex neotectonic history and the adverse climatic conditions of this area, both active at least since the Quaternary, resulted in the deep weathering of these crystalline rocks [32]. The short distance between the coast and the mountains, which rise to a maximum of nearly 1300 m above the sea level, resulted in the development of mountain torrents descending into highly braided and multi-channel streams [33]. Despite having little water for several months of the year and being almost totally dry during summer, Calabrian streams can become powerful to carry solid materials inshore resulting from up slope erosion to landslides [34].

In this context, the studied beach stretch represents the product of erosion, subsequent fluvial reworking and transport acted by rivers and tributaries. Incision and erosion rates are completely controlled by glacio-eustatic base-level fluctuations and by the huge uplift which involved this sector of the Calabrian Arc since the middle-late Quaternary, with rates up to ∼2 mm/year related to the activity of local normal faults added to the regional uplift [35].

Widespread in the studied area is the analysis of beach sands resulting from the disintegration of the source rock called “Cardinal tonalides”. They are part of the permo-carboniferous plutonic complex of the Serre and consist of the following units: “cardinal tonalites”, “granodiorites of Sant’Andrea Apostolo on the Ionian”, granodiorites, “granites of Isca on the Ionian Sea” and “leucogranites of Petrizzi” [36].

## 3. Materials and Methods

### 3.1. In Situ Radiometric Analysis

The environmental dose rate measurements were performed at 1 m height above ground level using a portable battery charged pressurized ion chamber survey meter, LUDLUM 9 DP (Ludlum Measurements, Inc., Sweetwater, TX, USA), 0–50 mSv/h dose rate range, 60 keV–1.25 MeV energy range [37].

All readings in the beach stretch, at a distance of 1 m from each other, were performed in the sand area through a linear transect that covered the region of interest. Ten readings of 600 s each, taken at each point of the transect, were carried out, and the average was recorded.

### 3.2. Sand Sampling and Preparation

All sand samples were collected at a depth of 0–15 cm from the spot characterized by the highest gamma dose rate determined by in situ measurement, for each municipality of the investigated beach stretch, according to [38]. Five samples, around 1 kg each, were collected for each sampling point by using a metal sampler and stored into labelled plastic boxes before being transported to the laboratory.

The sampling sites are reported in Table 1, together with their IDs and GPS coordinates.

**Figure 1:**
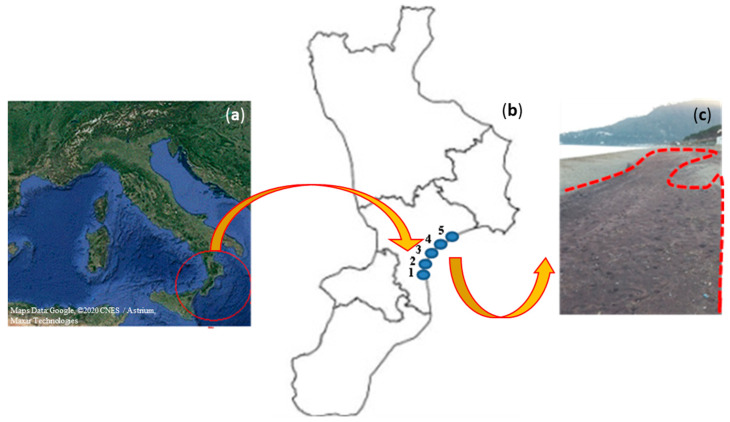
The map of the investigated area (**a**), with the site IDs (1–5) (**b**) and a typical image of the particular reddish sand (**c**) reported.

The map of the investigated area is shown in Figure 1, with the site IDs and a typical image of the particular reddish sand, characteristic of the investigated stretch of beach, reported.

### 3.3. Gamma Spectrometry Analysis

For the gamma spectrometry measurements, samples were dried at 105 °C in an oven, sieved to obtain a particle size less than 2 mm, then inserted in Marinelli hermetically sealed containers of 1 L capacity and left for a period of 40 days in order to reach the secular radioactive equilibrium between ^226^Ra and its daughter products. After that, the specific activity of ^226^Ra was quantified.

In our case, an acquisition time of 70,000 s was used. The 295.21 and 351.92 keV ^214^Pb and 1120.29 keV ^214^Bi gamma-ray lines were used to quantify the ^226^Ra specific activity. The ^232^Th activity concentration was determined by using the 911.21 and 968.97 keV ^228^Ac gamma-ray lines; it can be assumed that ^228^Ra, ^228^Ac and ^228^Th were in secular equilibrium with the parent nuclide ^232^Th, which is common in these sorts of soil samples [39,40]. Regarding ^40^K, the evaluation was performed from its γ-line at 1460.8 keV. In order to investigate the anthropogenic radioactivity content, the ^137^Cs specific activity was quantified through its γ-line at 661.66 keV.

Gamma spectrometry measurements were collected through two electrically-cooled Ortec HPGe detectors (Ametek Ortec, Oak Ridge, TN, USA) placed inside lead wells in order to shield the background radioactivity [41]. The first detector (GMX) is a reverse biased semiconductor having a 1.94 keV FWHM resolution, a 37.5% relative efficiency at the reference peak (^60^Co at 1.33 MeV) and a 65:1 peak to Compton ratio. The second detector (GEM) is a direct biased semiconductor characterized by 1.85 keV FWHM resolution, a 40% relative efficiency and a 64:1 peak to Compton ratio.

In order to perform efficiency and energy calibrations, Eckert and Zigler Nuclitec GmgH (Eckert & Ziegler GmbH Nuclitec, Braunschweig, Germany) traceable multinuclide radioactive standard, number AK 5901, covering the energy range 59.54 keV–1836 keV was employed. This calibration standard reproduced the exact geometries of samples in a water-equivalent epoxy resin matrix.

Experimental data were acquired and analysed with the Ortec Gamma Vision software [42].

For each identified radionuclide, the activity concentration was calculated as:

(1) $$C\;\left(Bq\;kg^{-1}\right)=\frac{N_{E}}{\varepsilon_{E}t\gamma_{d}M}$$

where *N_E_*, *ε_E_* and *γ_d_* account for the net area, the efficiency and yield of a photopeak at energy *E*, respectively; *M* is the dry mass (d.m.) of the sample (kg) and *t* is the acquisition time (s). Furthermore, counting statistics, nuclear data library, calibration efficiency, sample quantity, and self-absorption correction were considered for the evaluation of the combined standard measurement uncertainty at coverage factor k = 2.

In our case, the quality of the gamma spectrometry experimental results was certified by the Italian Accreditation Body (ACCREDIA) [43].

### 3.4. Evaluation of Radiological Indices

#### 3.4.1. Absorbed γ-Dose Rate

In order to assess the radiological health risk, the absorbed γ-dose rate (D) evaluation is the first major step. This radiological index was calculated as follows [1]:

D (nGy h^−1^) = 0.462*C_Ra_* + 0.604*C_Th_* + 0.0417*C_K_* (2)

where *C_Ra_, C_Th_*, and *C_K_* are the mean activity concentrations (Bq kg^−1^) of ^226^Ra, ^232^Th, and ^40^K in the analysed samples, respectively.

#### 3.4.2. The Annual Effective Dose Equivalent

The Annual Effective Dose Equivalent (AEDE) for an individual spending three months (during the summer period) in the investigated areas was quantified by using the conversion coefficient to effective dose of 0.7 Sv Gy^−1^ and the spending time in beach (432 h) [44]:

AEDE (outdoor) (µSv y^−1^) = absorbed dose (nGy h^−1^) × 432 h × 0.7 Sv Gy^−1^ × 10^−3^ (3)

### 3.5. X-ray Fluorescence

XRF measurements were collected by means of a benchtop Spectro Xepos spectrometer [45]. The instrument is equipped with a 50 W/60 kV W anode X-ray tube excitation source, and a highly sensitive and high resolution Silicon drift detector (SDD). The power and the current intensity were changed according to the analysed element and its quantity, in order to avoid the detector saturation.

For the XRF analysis, samples were prepared as follows: 5 g of finely powdered sediments, mixed with 2.6 g of wax, were placed in a 15 ton-press for 90 s to obtain a square tablet.

The concentrations of the elements were calculated through the use of the software package Turbo Quant II [45].

This method was validated using the standard sample IAEA-CU-2010-02 with which, by performing a quality control, the analysis must meet the narrow analytical range provided by the certificate of this standard.

### 3.6. Assessing the Level of Contamination of Heavy Metals

The level of contamination of heavy metals was assessed by employing some pollution indices such as the Enrichment Factor (EF), Geo-accumulation Index (I_geo_), Contamination Factor (CF) and Pollution Load Index (PLI).

#### 3.6.1. The Enrichment Factor

The Enrichment Factor is defined as:

(4) $$EF=\frac{\left\{C_{x}/C_{Fe}\right\}sample}{\left\{C_{x}/C_{Fe}\right\}reference},$$

where *C_x_* is the concentration of the potentially enrichment element and *C_Fe_* is the concentration of the normalizing element, usually Fe [46]. The world average elemental concentrations reported by [46] in the Earth’s crust were used as reference in this study being the regional geochemical background values for these elements not available.

#### 3.6.2. The Geoaccumulation Index

The Geoaccumulation Index was used to evaluate the degree of elemental pollution in the investigated samples, given by:

(5) $$I_{geo}=log_{2}\left[C_{n}/\left(kB_{n}\right)\right],$$

where *C_n_* is the concentration of the potentially hazardous trace element in the sample, *B_n_* is the geochemical background value in average shale [46] of the element *n* and *k* = 1.5 is the background matrix correction factor introduced to take into account possible differences in the background values due to lithogenic effects.

#### 3.6.3. The Contamination Factor

The Contamination Factor reflects the enrichment of heavy metals. It is the ratio of the concentration of a heavy metal in the investigated sample to the concentration of the same metal in the background source. CF is expressed as given below, as proposed by [47]:

(6) $$CF=C_{metal}/C_{background},$$

where *C_metal_* and *C_background_* are the concentration and the background values for each heavy metal, respectively.

#### 3.6.4. The Pollution Load Index

The Pollution Load Index provides a simple, comparative means for assessing the level of heavy metal pollution [48]. To evaluate the sample quality, an integrated approach of PLI of the detected heavy metals was calculated according to [49].

The PLI is the *n*-th root of the product of contamination factor (*CF*) of heavy metals, expressed as:

(7) PLI=(CF1×CF2×CF3×…×CFn)1n

where *n* is the number of metals.

### 3.7. X-ray Diffraction

The XRD analysis was performed using a Panalytical Empyrean Diffractometer with Cu K_α_ radiation on a Bragg-Brentano theta-theta goniometer, equipped with a solid-state detector, PIXcel [50].

The generator settings were 40 kV and 40 mA. The measurements were carried out in glass slide holders assuring a uniform distribution of suitably compressed sand samples. The 2θ incidence angle was spanned from 5° to 60° with a scan speed of 1.2° per minute using the continuous scan mode. The total runtime for each analysis was about 45 min.

To identify the crystalline mineral components of the investigated samples, the detected peak positions were compared with reference spectra from COD and RRUFF databases [51].

### 3.8. Micro-Raman Scattering

Micro-Raman Scattering was measured from different positions of several grains for each specimen, and the recorded spectra were compared with the library of the minerals with the higher abundance, as assessed by XRD analysis.

Raman scattering measurements were carried out using a HORIBA Scientific LabRAM HR Evolution Raman (Horiba Ltd., Kisshoin, Kyoto, Japan) spectrometer with an integrated Olympus BX41 (Olympus Corporation, Tokyo, Japan) microscope. A laser excitation wavelength of 532 nm (2.33 eV) was focused on the sample surface using an Olympus 50× objective (Olympus Corporation, Tokyo, Japan) with a spot size of approximately 2 μm. The acquisitions were performed on a minimum of 10 grains for each specimen, and the recorded spectra were compared with reference spectra obtained from the literature [52].

## 4. Results and Discussion

### 4.1. Radioactivity Analysis

The mean specific activity of detected natural and anthropogenic radionuclides, ^226^Ra, ^232^Th, ^40^K and ^137^Cs, in the investigated sand samples, is reported in Table 2.

In the case of radiocaesium, the average activity concentration was found to be lower than the minimum detectable activity in all cases, thus excluding an anthropogenic radioactive contamination of the investigated samples.

The highest activity concentration of ^226^Ra, ^232^Th and ^40^K was found in the site ID5 (for the first two radionuclides) and site ID2 (for the last one), respectively. Worldwide average concentrations of ^226^Ra, ^232^Th and ^40^K are 35, 30 and 400 Bq kg^−1^, respectively, as reported in the literature [1]. Experimental results show that, in our samples, the activity concentration of ^226^Ra is higher than the average world value only for the site ID5; for ^232^Th, it is higher than the worldwide one in all cases, except for the site ID2. The activity concentration of ^40^K is higher than the average world value for all investigated samples, except for the site ID5. It is worth to underline that the obtained values should be considered as strongly correlated with the geomorphological and the geographical features of the investigated site. Nevertheless, the activity concentration of natural radionuclides measured in this study were found to be in agreement with those obtained in other studies around the world [53,54,55,56], although such values change from one location to another depending on the geological setting.

### 4.2. Evaluation of Radiological Hazard Effects

#### 4.2.1. Absorbed Dose Rate

Calculated values of the absorbed dose rate, for the analysed samples, are reported in Table 3.

They are in the 51.2–351 nGy h^−1^ range, in the case of site ID2 and site ID5, respectively, with an average value (~160 nGy h^−1^) higher than the average world value (60 nGy h^−1^) [1].

**Figure 2:**
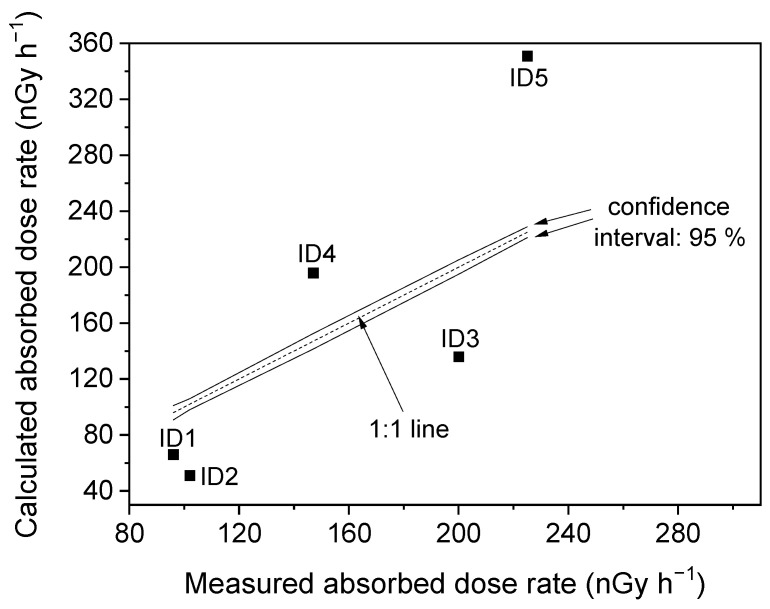
A comparison between calculated and measured absorbed dose rate.

In situ measured values (nGy h^−1^) of absorbed dose rate are also reported in Table 3. Obtained values vary between 96 nGy h^−1^ and 225 nGy h^−1^, with a mean value (~154 nGy h^−1^) higher than the average world value also in this case. In our case, measured values are outside the 95% confidence interval, as reported in Figure 2, indicating that the field and laboratory measurements are not mutually corroborative.

This is not surprising, considering that no good agreement between calculated and measured gamma-ray activity is usually found in the literature [57], due to the fact that measured values may be influenced by soil moisture, wash-out of dust containing radon decay products, and principally by radionuclide inhomogeneity in the soil.

#### 4.2.2. AEDE

Values of the AEDE are reported in Table 3. They are in the 15–106 µSv y^−1^ range, for site ID2 and site ID5, respectively, lower than the world average value of 70 µSv y^−1^ [58] in all cases, except for the Squillace sampling point (ID5), and much lower than the action levels provided by the Italian legislation (0.3 mSv y^−1^) for the population members [59].

Obtained results thus confirm that there are no hazard effects for the local population, from a radiological point of view.

### 4.3. Heavy Metals Analysis

Table 4 reports heavy metals content (µg g^−1^ d.w.) for the analysed sands, as obtained through XRF analysis.

In particular, Sb, As, Cd and Hg contents turned out to be lower than the minimum detectable concentration in all samples.

For all detected metals, the obtained concentrations are lower than the contamination thresholds values given by [32] and also reported in Table 4. Therefore, they cannot be considered as pollutants. As a consequence, they do not cause objectionable effects and do not impair the welfare of the environment. For this reason, they do not constitute a risk to human health. Obtained results are highly affected by the geochemical properties of the site. Nevertheless, the heavy metals concentrations found in this study turned out to be comparable with values typically reported in the literature [60,61,62,63].

### 4.4. Estimation of the Level of Heavy Metals Contamination

#### 4.4.1. EF

Obtained EF values are reported in Table 5, for the assessed heavy metals with a content value higher than the XRF minimum detectable concentration.

The EF values were interpreted as described by [64], where EF < 2 indicates deficient to minimal enrichment; 2 ≤ EF < 5 moderate enrichment; 5 ≤ EF < 20 significant enrichment; 20 ≤ EF ≤ 40 high enrichment and EF > 40 indicates extremely high enrichment.

Moreover, according to the literature [65], EF values between 0.5 and 1.5 indicate that the metal is entirely from crustal materials or natural origin, while EF > 1.5 suggests that the sources are more likely to be anthropogenic.

In our case, the obtained results showed that EF values for Co, Ni, Cu, V and Zn are lower than 2 for all investigated sites, indicating no or minimal enrichment. Regarding Pb, its EF value is lower than 2 only for the site ID5, whereas in the other sites a moderate enrichment is observed. This can be easily justified taking into account several factors related to anthropogenic contribution, such as exhaust fumes from motor-vehicle, smelting activities, indiscriminate dumping of used lead acid batteries, etc. Interestingly, regarding Tl, an EF value higher than 2 is obtained in the case of sampling site ID 4, thus highlighting for this area a moderate enrichment of Tl due to anthropic contribution.

#### 4.4.2. I_geo_

Obtained I_geo_ values for the heavy metals of the investigated beach stretch are reported in Table 5.

The I_geo_ values were interpreted as reported in the literature [66], according to which I_geo_ ≤ 0 means no contamination; 0 < I_geo_ ≤ 1 no/moderate contamination; 1 < I_geo_ ≤ 2 moderate contamination; 2 < I_geo_ ≤ 3 moderate/strong contamination; 3 < I_geo_ ≤ 4 strong contamination; 4 < I_geo_ ≤ 5 strong/extreme contamination and I_geo_ > 5 extreme contamination.

All values are < 0, indicating that no contamination is present for the investigated heavy metals.

#### 4.4.3. CF

CF values of heavy metals in the investigated samples are presented in Table 5.

They were interpreted as described by [67], according to which CF ≤ 1 indicates no contamination, 1 < CF ≤ 3 low or moderate contamination, 3 < CF ≤ 6 high contamination, CF > 6 very high contamination.

All values are <1, again indicating that no contamination is present for the investigated heavy metals.

#### 4.4.4. PLI

Table 5 reports the PLI values of the sampling points for the detected heavy metals of investigated sand samples.

As reported by [68], the PLI value > 1 implies pollution presence, whereas PLI value < 1 indicates no pollution.

For all investigated sites, PLI value is < 1. This result thus reveals that all the sampling points are not polluted by assessed heavy metals.

### 4.5. XRD Analysis

**Figure 3:**
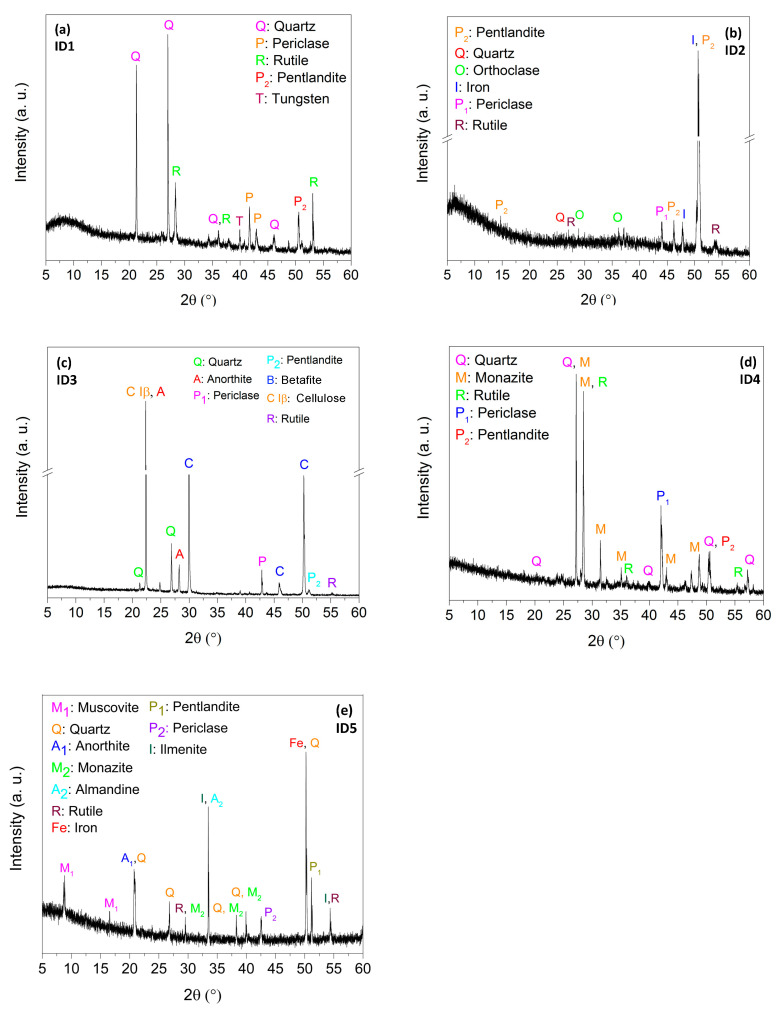
X-ray diffraction spectra of samples ID# (# = 1, …, 5) (**a**–**e**).

The X-ray diffraction analyses are shown in Figure 3. Minerals’ identification was carried out by matching the measured diffraction peak positions to the COD and RRUFF databases. XRD analyses show that all the samples were characterized by the presence of Quartz (SiO_2_, Ref. COD: 96-901-2602), Periclase (MgO, two phases: Ref. COD: 96-900-6470 and 96-901-3218), Pentlandite ((Ni, Fe)_9_S_8_, Ref. COD: 96-901-0107) and Rutile (TiO_2_, RRUFF ID: R040049).

Iron (Fe, Ref. COD: 96-900-6658) was found in great quantity in samples ID2 and ID5. Monazite ((Ce, La, Pr, Nd, Th, Y)PO_4_, RRUFF ID: R040106) was the most abundant mineral in sample ID4 and it was also found in sample ID5. Sample ID3 contained a large amount of cellulose (C Iβ, C_12_H_14_O_10_ [69]), betafite (Ca_2_(Nb,Ti)_2_(O,OH)_7_, Ref. COD: 96-900-0873) and Anorthite (Ca(Al_2_Si_2_O_8_), RRUFF ID: R040059). Muscovite (KAl_2_(Si_3_Al)O_10_(OH)_2_, RRUFF ID: R040104), Anorthite (Ca(Al_2_Si_2_O_8_), RRUFF ID: R040059), Almandine (Fe_3_Al_2_(SiO_4_)_3_, RRUFF ID: R120145) and Ilmenite (FeTiO_3_, RRUFF ID: R060149) were present in sample ID5. Finally, contents of orthoclase (KAlSi_3_O_8_, RRUFF ID: R040055) and tungsten (W, Ref. COD: 96-900-6498) were found in sample ID2 and ID1, respectively. These findings are in agreement with the results emerging from the XRF analysis.

### 4.6. MRS Analysis

**Figure 4:**
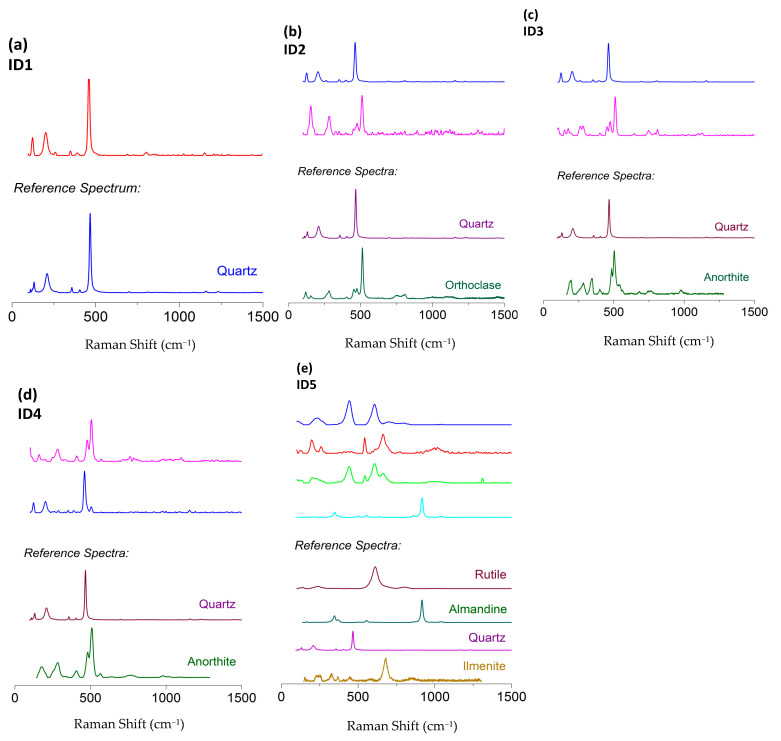
Micro-Raman scattering measurements of samples ID# (# = 1, …, 5) (**a**–**e**).

Micro-Raman scattering measurements are displayed in Figure 4. The most representative spectra acquired on each sample are compared to literature references.

Quartz (RRUFF ID: R040031) was clearly identified in all the investigated samples. Together with quartz, orthoclase (RRUFF ID: R040055) fingerprint was detected in sample ID2, while the Raman signals of anorthite (RRUFF ID: R040059) were found in samples ID3 and ID4. Finally, the Raman analysis carried out on sample ID5 confirmed the presence of rutile (RRUFF ID: R040049), almandine (RRUFF ID: R120145) and ilmenite (RRUFF ID: R060149). Such results validate the XRD analysis. It is important to mention that the laser spot size is about 2 μm, therefore it is not straightforward to obtain the Raman fingerprints of all the minerals present in each sample.

## 5. Conclusions

The pollution levels of natural and anthropogenic radionuclides and heavy metals of the beach stretch going from the Soverato to the Squillace municipalities, in the Ionian coast of Calabria, south of Italy, were investigated through High Purity Germanium (HPGe) gamma-ray and X-ray Fluorescence (XRF) spectrometry.

Calculations of the absorbed dose rate and of the annual effective dose equivalent were performed to assess any possible radiological hazard for tourists or the inhabitant population. Values of the AEDE are much lower than the action levels provided by the Italian legislation (0.3 mSv y^−1^) for the population members, then confirming no hazard effects for the local population, from the radiological point of view.

The heavy metals ecological risk imposed on the ecosystems was assessed through the calculation of different pollution indices, such as enrichment factor (EF), geo-accumulation index (I_geo_), contamination factor (CF) and pollution load index (PLI). In particular, EF values of Co, Ni, Cu, Tl, V and Zn for all investigated sites indicate no or minimal enrichment. An anthropogenic contribution was suggested by the moderate enrichment of Pb, regarding sites ID1-4, and of Tl, as far as site ID4 is concerned. Furthermore, the obtained I_geo_, CF and PLI values indicated that no contamination due to the investigated metals is present.

In order to correlate the radioactivity emission and the heavy metals content to the mineralogical and geochemical characterization of the investigated beach stretch, a mineralogical analysis was performed through X-ray Diffractometry (XRD) and Micro-Raman Scattering (MRS) spectroscopy. From the results, the main role of monazite in increasing the ^232^Th radioactivity concentration can be hypothesized.

## Figures and Tables

**Table 1:** The sampling sites, together with their IDs and GPS coordinates.

Site ID	Sampling Site	GPS Position	
		Latitude	Longitude
1	Soverato	38°41′16′′ N	16°33′20′′ E
2	Montepaone	38°43′17′′ N	16°32′17′′ E
3	Montauro	38°44′07′′ N	16°33′07′′ E
4	Stalettì	3846′ 08′′ N	16°34′10′′ E
5	Squillace	38°46′40′′ N	16°34′26′′ E

**Table 2:** The mean specific activity of ^226^Ra, ^232^Th, ^40^K and ^137^Cs, in the investigated samples.

Site ID	^226^Ra (Bq kg^−1^ d.w.)	^232^Th (Bq kg^−1^ d.w.)	^40^K (Bq kg^−1^ d.w.)	^137^Cs (Bq kg^−1^ d.w.)
1	8.3 ± 0.8	57.9 ± 7.6	656 ± 84	<0.15
2	7.9 ± 0.8	26.3 ± 3.5	759 ± 98	<0.11
3	12.3 ± 1.3	183 ± 24	469 ± 60	<0.31
4	15.8 ± 1.6	277 ± 36	505 ± 65	<0.15
5	60.2 ± 6.4	524 ± 73	148 ± 20	<0.30

**Table 3:** Calculated and measured values of the absorbed dose rate, and AEDE, for the analysed samples.

Site ID	Calculated Absorbed Dose Rate (nGy h^−1^)	Measured Absorbed Dose Rate (nGy h^−1^)	AEDE (µSv y^−1^)
1	66.2	96	20
2	51.2	102	15
3	136	200	41
4	196	147	59
5	351	225	106

**Table 4:** Heavy metals content (µg g^−1^ d.w.) for the analysed sands, as obtained through XRF analysis.

XRF Analysis						
	Site ID					Threshold Limit
	1	2	3	4	5	
Sb (µg g^−1^ d.w.)	<0.5	<0.5	<0.5	<0.5	<0.5	10
As (µg g^−1^ d.w.)	<0.5	<0.5	<0.5	<0.5	<0.5	20
Cd (µg g^−1^ d.w.)	<0.3	<0.2	<0.3	<0.3	<0.7	2
Co (µg g^−1^ d.w.)	3.0 ± 0.1	<3.0	<3.0	<2.1	18.8 ± 5.4	20
Hg (µg g^−1^ d.w.)	<0.7	<0.7	<0.7	<0.7	<0.7	1
Ni (µg g^−1^ d.w.)	3.4 ± 0.8	3.4 ± 0.7	3.4 ± 0.7	5.7 ± 0.8	<0.5	120
Pb (µg g^−1^ d.w.)	13.6 ± 0.4	12.8 ± 0.4	10.7 ± 0.4	11.4 ± 0.4	12.5 ± 1.1	100
Cu (µg g^−1^ d.w.)	1.8 ± 0.6	<0.7	2.1 ± 0.6	1.2 ± 0.6	4.9 ± 1.8	120
Tl (µg g^−1^ d.w.)	<0.7	<0.7	<0.7	0.8 ± 0.3	<0.7	1
V (µg g^−1^ d.w.)	11.9 ± 1.3	3.6 ± 0.5	11.9 ± 1.1	5.2 ± 0.4	<1.0	90
Zn (µg g^−1^ d.w.)	15.4 ± 0.5	14.3 ± 0.5	15.0 ± 0.5	15.7 ± 0.5	73.0 ± 2.0	150

**Table 5:** Calculated values of the enrichment factor (EF), geoaccumulation index (I_geo_), contamination factor (CF) and pollution load index (PLI) for all the investigated sites.

Site ID	Metal	Index of Contamination			
		EF	I_geo_	CF	PLI
1	Co	0.76	−3.25	0.16	0.05
	Ni	0.24	−4.91	0.05	
	Pb	3.26	−1.14	0.68	
	Cu	0.19	−5.23	0.04	
	Tl	-	-	-	
	V	0.44	−4.03	0.09	
	Zn	0.78	−3.21	0.16	
2	Co	-	-	-	0.08
	Ni	0.37	−4.91	0.05	
	Pb	4.77	−1.23	0.64	
	Cu	-	-	-	
	Tl	-	-	-	
	V	0.21	−5.76	0.03	
	Zn	1.12	−3.32	0.15	
3	Co	-	-	-	0.07
	Ni	0.22	−4.91	0.05	
	Pb	2.39	−1.49	0.54	
	Cu	0.21	−5.01	0.05	
	Tl	-	-	-	
	V	0.41	−4.03	0.09	
	Zn	0.71	−3.25	0.16	
4	Co	-	-	-	0.06
	Ni	0.38	−4.16	0.08	
	Pb	2.60	−1.40	0.57	
	Cu	0.12	−5.81	0.03	
	Tl	4.24	−0.69	0.93	
	V	0.18	−5.23	0.04	
	Zn	0.75	−3.18	0.17	
5	Co	0.49	−0.45	1.09	0.58
	Ni	-	-	-	
	Pb	0.28	−1.26	0.63	
	Cu	0.05	−3.78	0.11	
	Tl	-	-	-	
	V	-	-	-	
	Zn	0.34	−0.96	0.77	

## Data Availability

Data sharing is not applicable to this article.
